# Supplementary material for: Five Post-Translational Modification Residues of CmPT2 Play Key Roles in Yeast and Rice
Source: Int J Mol Sci. 2023 Jan 19;24(3):2025. doi: 10.3390/ijms24032025 (PMC9953561; doi:10.3390/ijms24032025)
Supplement: Supplementary file 1 [file ijms-24-02025-s001.zip › supplementary figures.pdf]

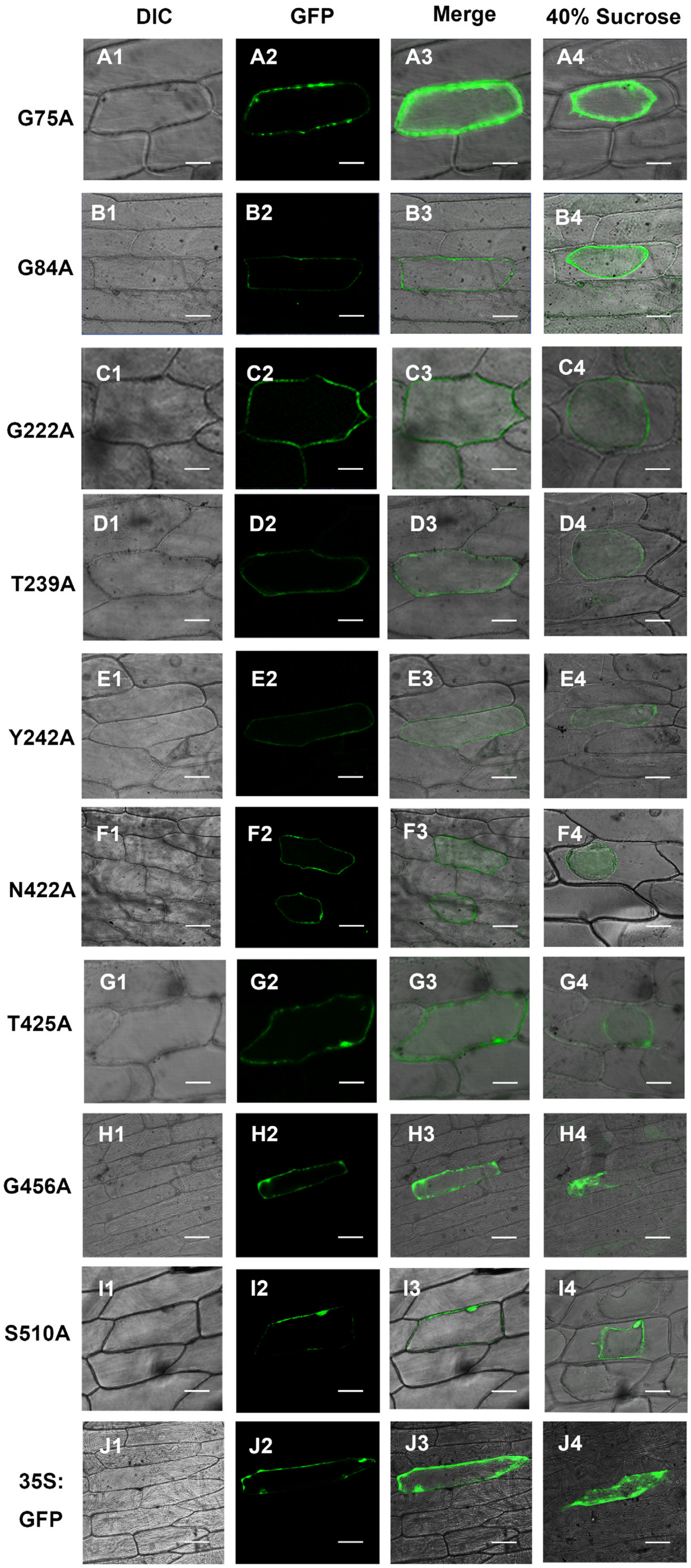
DIC
GFP
Merge
40% Sucrose
G75A
G84A
G222A
T239A
Y242A
N422A
T425A
G456A
S510A
35S:
GFP
A1
A2
A3
A4
B1
B2
B3
B4
C1
C2
C3
C4
D1
D2
D3
D4
E1
E2
E3
E4
F1
F2
F3
F4
G1
G2
G3
G4
H1
H2
H3
H4
I1
I2
I3
I4
J1
J2
J3
J4

Supplementary Figure S1. Subcellular localization of Cmphts. A1-I1: cytoarchitecture; A2-I2: fluorescence signal; A3-I3: overlay plots; A4-I4: plasmolysis (40% Sucrose), Bar: 50 μm.

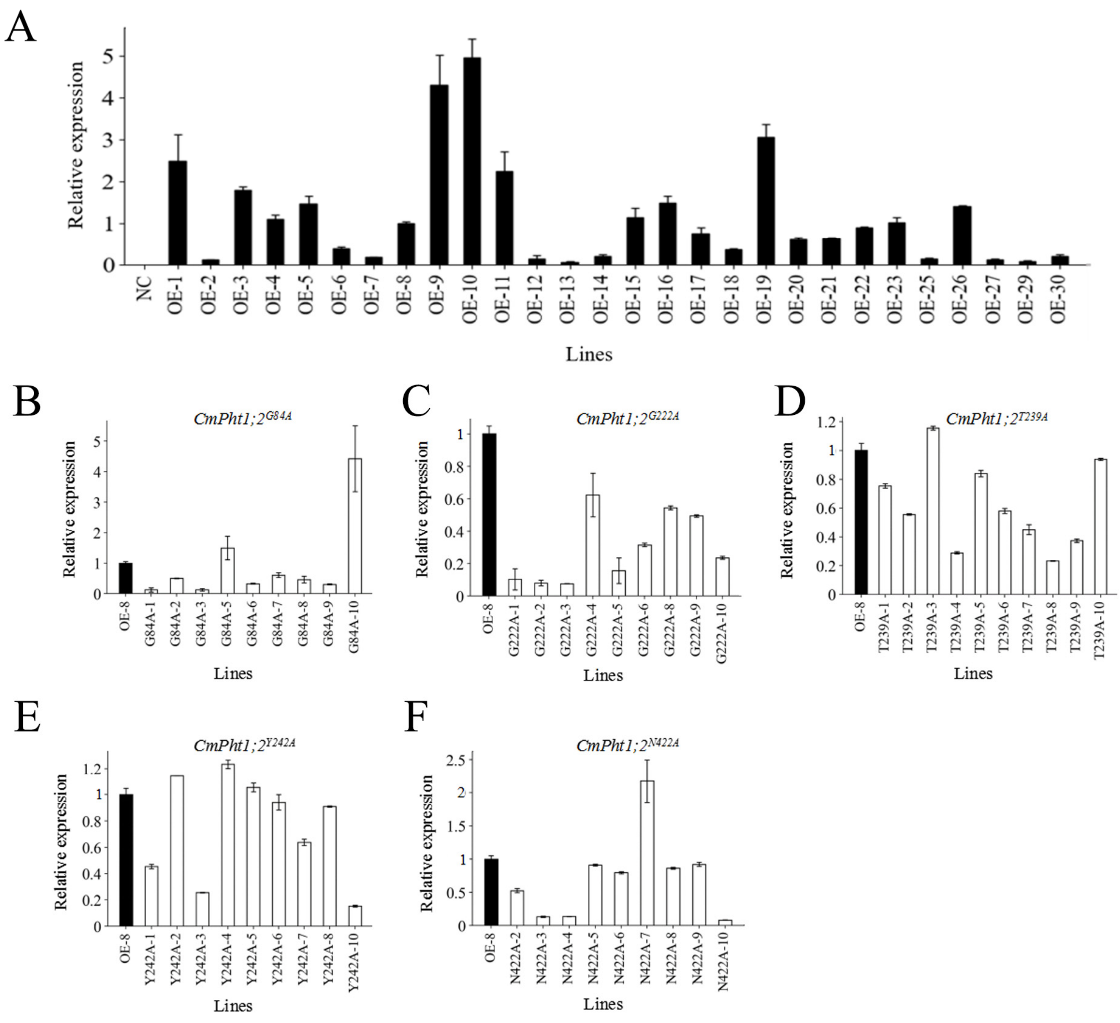

Supplementary Figure S2. Relative expression analysis of CmPT2 between W7 and CmPT2-OE lines (A), G84A-OE lines (B), G222A-OE lines (C), T239A-OE lines (D), Y242A-OE lines (E), N422A-OE lines (F).
