# Supplementary figures and images for: Five Post-Translational Modification Residues of CmPT2 Play Key Roles in Yeast and Rice
Source: Int J Mol Sci. 2023 Jan 19;24(3):2025. doi: 10.3390/ijms24032025 (PMC9953561; doi:10.3390/ijms24032025)

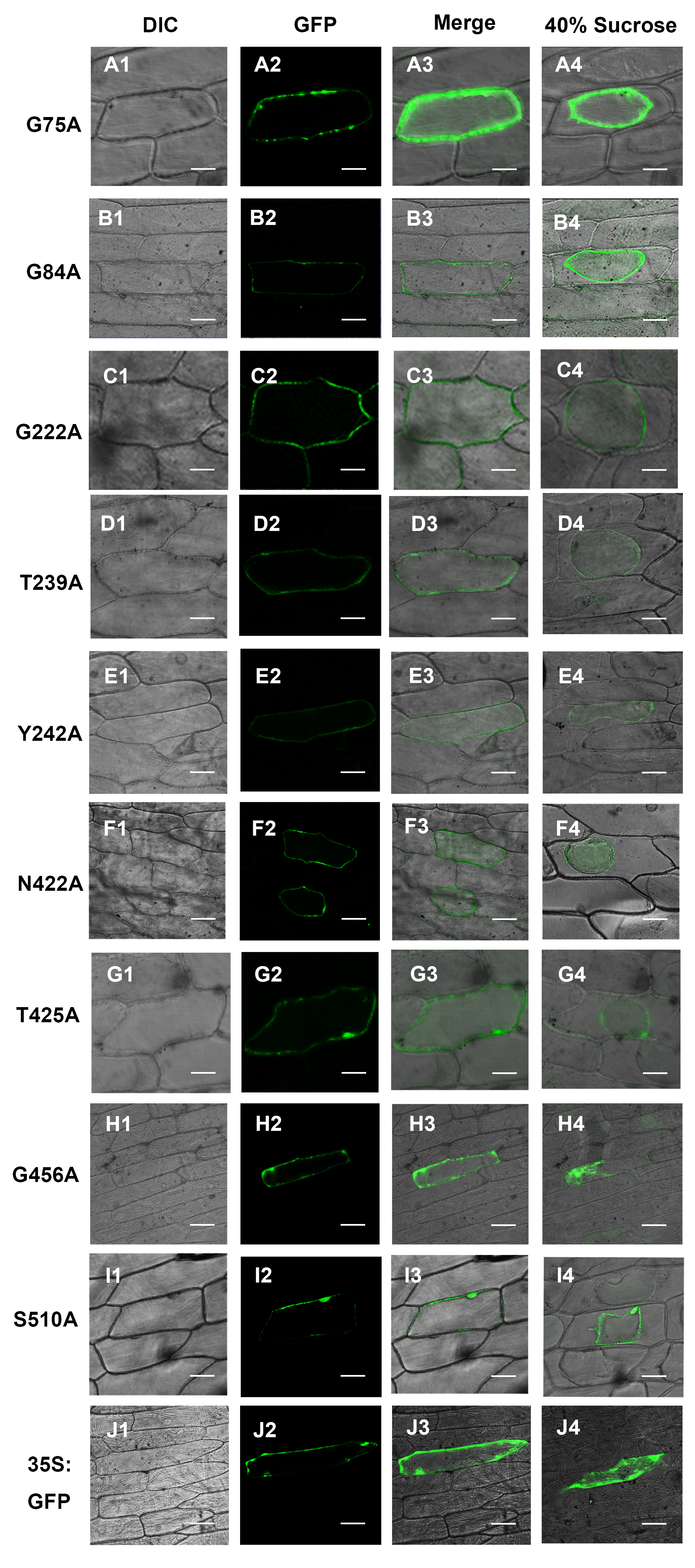

Supplement: Supplementary file 1 [file ijms-24-02025-s001.zip › Supplementary Figure S1.tif]

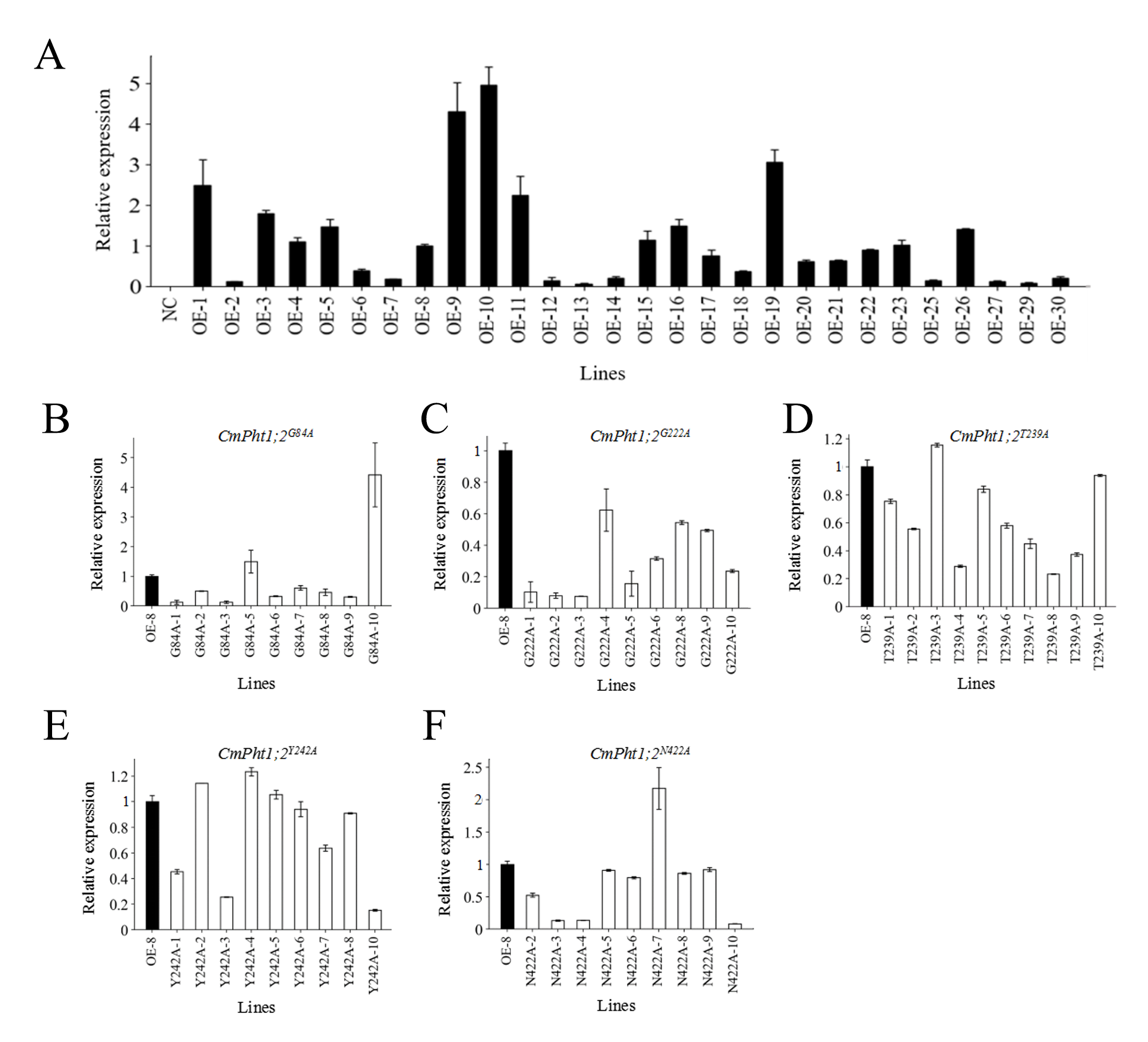

Supplement: Supplementary file 1 [file ijms-24-02025-s001.zip › Supplementary Figure S2.tif]
